# The predictive ability of the 313 variant–based polygenic risk score for contralateral breast cancer risk prediction in women of European ancestry with a heterozygous *BRCA1* or *BRCA2* pathogenic variant

**DOI:** 10.1038/s41436-021-01198-7

**Published:** 2021-06-10

**Authors:** Inge M. M. Lakeman, Alexandra J. van den Broek, Juliën A. M. Vos, Daniel R. Barnes, Julian Adlard, Irene L. Andrulis, Adalgeir Arason, Norbert Arnold, Banu K. Arun, Judith Balmaña, Daniel Barrowdale, Javier Benitez, Ake Borg, Trinidad Caldés, Maria A. Caligo, Wendy K. Chung, Kathleen B. M. Claes, GEMO Study Collaborators, Emmanuelle Barouk-Simonet, Muriel Belotti, Pascaline Berthet, Yves-Jean Bignon, Valérie Bonadona, Brigitte Bressac-de Paillerets, Bruno Buecher, Sandrine Caputo, Olivier Caron, Laurent Castera, Virginie Caux-Moncoutier, Chrystelle Colas, Marie-Agnès Collonge-Rame, Isabelle Coupier, Antoine de Pauw, Capucine Delnatte, Camille Elan, Laurence Faivre, Sandra Fert Ferrer, Marion Gauthier-Villars, Paul Gesta, Sophie Giraud, Lisa Golmard, Claude Houdayer, Christine Lasset, Maïté Laurent, Dominique Leroux, Michel Longy, Véronique Mari, Sylvie Mazoyer, Noura Mebirouk, Isabelle Mortemousque, Fabienne Prieur, Pascal Pujol, Claire Saule, Helene Schuster, Nicolas Sevenet, Hagay Sobol, Johanna Sokolowska, Laurence Venat-Bouvet, EMBRACE Collaborators, Munaza Ahmed, Julian Barwell, Angela Brady, Paul Brennan, Carole Brewer, Jackie Cook, Rosemarie Davidson, Alan Donaldson, Alison M. Dunning, Jacqueline Eason, Diana M. Eccles, Helen Gregory, Helen Hanson, Patricia A. Harrington, Alex Henderson, Shirley Hodgson, M. John Kennedy, Fiona Lalloo, Clare Miller, Patrick J. Morrison, Kai-ren Ong, Aoife O’Shaughnessy-Kirwan, Jo Perkins, Mary E. Porteous, Mark T. Rogers, Lucy E. Side, Katie Snape, Lisa Walker, J. Margriet Collée, Fergus J. Couch, Mary B. Daly, Joe Dennis, Mallika Dhawan, Susan M. Domchek, Ros Eeles, Christoph Engel, D. Gareth Evans, Lidia Feliubadaló, Lenka Foretova, Eitan Friedman, Debra Frost, Patricia A. Ganz, Judy Garber, Simon A. Gayther, Anne-Marie Gerdes, Andrew K. Godwin, David E. Goldgar, Eric Hahnen, Christopher R. Hake, Ute Hamann, Frans B. L. Hogervorst, Maartje J. Hooning, John L. Hopper, Peter J. Hulick, Evgeny N. Imyanitov, OCGN Investigators, Gord Glendon, Anna Marie Mulligan, HEBON Investigators, Christi J. van Asperen, Cora M. Aalfs, Muriel A. Adank, Margreet G. E. M. Ausems, Marinus J. Blok, Encarna B. Gómez Garcia, Bernadette A. M. Heemskerk-Gerritsen, Antoinette Hollestelle, Agnes Jager, Linetta B. Koppert, Marco Koudijs, Mieke Kriege, Hanne E. J. Meijers-Heijboer, Arjen R. Mensenkamp, Thea M. Mooij, Jan C. Oosterwijk, Ans M. W. van den Ouweland, Frederieke H. van der Baan, Annemieke H. van der Hout, Lizet E. van der Kolk, Rob B. van der Luijt, Carolien H. M. van Deurzen, Helena C. van Doorn, Klaartje van Engelen, Liselotte P. van Hest, Theo A. M. van Os, Senno Verhoef, Maartje J. Vogel, Juul T. Wijnen, KconFab Investigators, Jonathan Beesley, Stephen Fox, Helene Holland, Kelly-Anne Phillips, Amanda B. Spurdle, Claudine Isaacs, Louise Izatt, Anna Jakubowska, Paul A. James, Ramunas Janavicius, Uffe Birk Jensen, Yue Jiao, Esther M. John, Vijai Joseph, Beth Y. Karlan, Carolien M. Kets, Irene Konstantopoulou, Ava Kwong, Clémentine Legrand, Goska Leslie, Fabienne Lesueur, Jennifer T. Loud, Jan Lubiński, Siranoush Manoukian, Lesley McGuffog, Austin Miller, Denise Molina Gomes, Marco Montagna, Emmanuelle Mouret-Fourme, Katherine L. Nathanson, Susan L. Neuhausen, Heli Nevanlinna, Joanne Ngeow Yuen Yie, Edith Olah, Olufunmilayo I. Olopade, Sue K. Park, Michael T. Parsons, Paolo Peterlongo, Marion Piedmonte, Paolo Radice, Johanna Rantala, Gad Rennert, Harvey A. Risch, Rita K. Schmutzler, Priyanka Sharma, Jacques Simard, Christian F. Singer, Zsofia Stadler, Dominique Stoppa-Lyonnet, Christian Sutter, Yen Yen Tan, Manuel R. Teixeira, Soo Hwang Teo, Alex Teulé, Mads Thomassen, Darcy L. Thull, Marc Tischkowitz, Amanda E. Toland, Nadine Tung, Elizabeth J. van Rensburg, Ana Vega, Barbara Wappenschmidt, Peter Devilee, Christi J. van Asperen, Jonine L. Bernstein, Kenneth Offit, Douglas F. Easton, Matti A. Rookus, Georgia Chenevix-Trench, Antonis C. Antoniou, Mark Robson, Marjanka K. Schmidt

**Affiliations:** 1 Department of Human Genetics, Leiden University Medical Center, Leiden, The Netherlands; 2 Department of Clinical Genetics, Leiden University Medical Center, Leiden, The Netherlands; 3 Division of Molecular Pathology, The Netherlands Cancer Institute–Antoni van Leeuwenhoek Hospital, Amsterdam, The Netherlands; 4 Centre for Cancer Genetic Epidemiology, Department of Public Health and Primary Care, University of Cambridge, Cambridge, UK; 5 Yorkshire Regional Genetics Service, Chapel Allerton Hospital, Leeds, UK; 6 Fred A. Litwin Center for Cancer Genetics, Lunenfeld-Tanenbaum Research Institute of Mount Sinai Hospital, Toronto, ON Canada; 7 Department of Molecular Genetics, University of Toronto, Toronto, ON Canada; 8 Department of Pathology, Landspitali University Hospital, Reykjavik, Iceland; 9 BMC (Biomedical Centre), Faculty of Medicine, University of Iceland, Reykjavik, Iceland; 10 Department of Gynaecology and Obstetrics, University Hospital of Schleswig-Holstein, Campus Kiel, Christian-Albrechts University Kiel, Kiel, Germany; 11 Institute of Clinical Molecular Biology, University Hospital of Schleswig-Holstein, Campus Kiel, Christian-Albrechts University Kiel, Kiel, Germany; 12 Department of Breast Medical Oncology, University of Texas MD Anderson Cancer Center, Houston, TX USA; 13 Hereditary cancer Genetics Group, Vall d’Hebron Institute of Oncology, Barcelona, Spain; 14 Department of Medical Oncology, Vall d’Hebron Barcelona Hospital Campus, University Hospital of Vall d’Hebron, Barcelona, Spain; 15 Centro de Investigación en Red de Enfermedades Raras (CIBERER), Madrid, Spain; 16 Human Cancer Genetics Programme, Spanish National Cancer Research Centre (CNIO), Madrid, Spain; 17 Department of Oncology, Lund University and Skåne University Hospital, Lund, Sweden; 18 Molecular Oncology Laboratory, CIBERONC, Hospital Clinico San Carlos, IdISSC (Instituto de Investigación Sanitaria del Hospital Clínico San Carlos), Madrid, Spain; 19 SOD Genetica Molecolare. University Hospital, Pisa, Italy; 20 Departments of Pediatrics and Medicine, Columbia University, New York, NY USA; 21 Centre for Medical Genetics, Ghent University, Ghent, Belgium; 22 Department of Clinical Genetics, Erasmus University Medical Center, CA Rotterdam, The Netherlands; 23 Department of Laboratory Medicine and Pathology, Mayo Clinic, Rochester, MN USA; 24 Department of Clinical Genetics, Fox Chase Cancer Center, Philadelphia, PA USA; 25 Cancer Genetics and Prevention Program, University of California San Francisco, San Francisco, CA USA; 26 Basser Center for BRCA, Abramson Cancer Center, University of Pennsylvania, Philadelphia, PA USA; 27 Oncogenetics Team, The Institute of Cancer Research and Royal Marsden NHS Foundation Trust, London, UK; 28 Institute for Medical Informatics, Statistics and Epidemiology, University of Leipzig, Leipzig, Germany; 29 Division of Evolution and Genomic Sciences, School of Biological Sciences, Faculty of Biology, Medicine and Health, University of Manchester, Manchester Academic Health Science Centre, Manchester, UK; 30 North West Genomics Laboratory Hub, Manchester Centre for Genomic Medicine, St Mary’s Hospital, Manchester University NHS Foundation Trust, Manchester Academic Health Science Centre, Manchester, UK; 31 Hereditary Cancer Program, ONCOBELL-IDIBELL-IGTP, Catalan Institute of Oncology, CIBERONC, Barcelona, Spain; 32 Department of Cancer Epidemiology and Genetics, Masaryk Memorial Cancer Institute, Brno, Czech Republic; 33 The Susanne Levy Gertner Oncogenetics Unit, Chaim Sheba Medical Center, Ramat Gan, Israel; 34 Sackler Faculty of Medicine, Tel Aviv University, Ramat Aviv, Israel; 35 Schools of Medicine and Public Health, Division of Cancer Prevention & Control Research, Jonsson Comprehensive Cancer Centre, UCLA, Los Angeles, CA USA; 36 Cancer Risk and Prevention Clinic, Dana-Farber Cancer Institute, Boston, MA USA; 37 Center for Bioinformatics and Functional Genomics and the Cedars Sinai Genomics Core. Cedars-Sinai Medical Center, Los Angeles, CA USA; 38 Department of Clinical Genetics, Rigshospitalet, Copenhagen University Hospital, Copenhagen, Denmark; 39 Department of Pathology and Laboratory Medicine, University of Kansas Medical Center, Kansas City, KS USA; 40 Department of Dermatology, Huntsman Cancer Institute, University of Utah School of Medicine, Salt Lake City, UT USA; 41 Center for Familial Breast and Ovarian Cancer, Faculty of Medicine and University Hospital Cologne, University of Cologne, Cologne, Germany; 42 Center for Integrated Oncology (CIO), Faculty of Medicine and University Hospital Cologne, University of Cologne, Cologne, Germany; 43 Waukesha Memorial Hospital-Pro Health Care, Waukesha, WI USA; 44 Molecular Genetics of Breast Cancer, German Cancer Research Center (DKFZ), Heidelberg, Germany; 45 Family Cancer Clinic, The Netherlands Cancer Institute–Antoni van Leeuwenhoek Hospital, Amsterdam, The Netherlands; 46 Department of Medical Oncology, Erasmus MC Cancer Institute, Rotterdam, The Netherlands; 47 Centre for Epidemiology and Biostatistics, Melbourne School of Population and Global Health, The University of Melbourne, Melbourne, VIC Australia; 48 Center for Medical Genetics, NorthShore University HealthSystem, Evanston, IL USA; 49 The University of Chicago Pritzker School of Medicine, Chicago, IL USA; 50 N.N. Petrov Institute of Oncology, St. Petersburg, Russia; 51 Lombardi Comprehensive Cancer Center, Georgetown University, Washington, DC USA; 52 Clinical Genetics, Guy’s and St Thomas’ NHS Foundation Trust, London, UK; 53 Department of Genetics and Pathology, Pomeranian Medical University, Szczecin, Poland; 54 Independent Laboratory of Molecular Biology and Genetic Diagnostics, Pomeranian Medical University, Szczecin, Poland; 55 Parkville Familial Cancer Centre, Peter MacCallum Cancer Center, Melbourne, VIC Australia; 56 Sir Peter MacCallum Department of Oncology, The University of Melbourne, Melbourne, VIC Australia; 57 Hematology, Oncology and Transfusion Medicine Center, Department of Molecular and Regenerative Medicine, Vilnius University Hospital Santariskiu Clinics, Vilnius, Lithuania; 58 State Research Institute Centre for Innovative Medicine, Vilnius, Lithuania; 59 Department of Clinical Genetics, Aarhus University Hospital, Aarhus, Denmark; 60 Genetic Epidemiology of Cancer team, Paris, France; 61 Institut Curie, Paris, France; 62 Mines ParisTech, Fontainebleau, France; 63 Department of Epidemiology & Population Health, Stanford University School of Medicine, Stanford, CA USA; 64 Department of Medicine, Division of Oncology, Stanford Cancer Institute, Stanford University School of Medicine, Stanford, CA USA; 65 Clinical Genetics Research Lab, Department of Cancer Biology and Genetics, Memorial Sloan Kettering Cancer Center, New York, NY USA; 66 David Geffen School of Medicine, Department of Obstetrics and Gynecology, University of California at Los Angeles, Los Angeles, CA USA; 67 Molecular Diagnostics Laboratory, INRASTES, National Centre for Scientific Research ‘Demokritos’, Athens, Greece; 68 Hong Kong Hereditary Breast Cancer Family Registry, Cancer Genetics Centre, Happy Valley, Hong Kong; 69 Department of Surgery, The University of Hong Kong, Pok Fu Lam, Hong Kong; 70 Department of Surgery, Hong Kong Sanatorium and Hospital, Happy Valley, Hong Kong; 71 Département de Génétique, CHU de Grenoble, Grenoble, France; 72 Clinical Genetics Branch, Division of Cancer Epidemiology and Genetics, National Cancer Institute, Bethesda, MD USA; 73 Unit of Medical Genetics, Department of Medical Oncology and Hematology, Fondazione IRCCS Istituto Nazionale dei Tumori di Milano, Milan, Italy; 74 NRG Oncology, Statistics and Data Management Center, Roswell Park Cancer Institute, Buffalo, NY USA; 75 Service de Biologie de la reproduction, Cytogénétique et Génétique Médicale, CHI Poissy–Saint Germain, Poissy, France; 76 Immunology and Molecular Oncology Unit, Veneto Institute of Oncology IOV–IRCCS, Padua, Italy; 77 Service de Génétique, Institut Curie, Paris, France; 78 Department of Population Sciences, Beckman Research Institute of City of Hope, Duarte, CA USA; 79 Department of Obstetrics and Gynecology, Helsinki University Hospital, University of Helsinki, Helsinki, Finland; 80 Cancer Genetics Service, National Cancer Centre, Singapore, Singapore; 81 Lee Kong Chian School of Medicine, Nanyang Technological University, Singapore, Singapore; 82 Department of Molecular Genetics, National Institute of Oncology, Budapest, Hungary; 83 Center for Clinical Cancer Genetics, The University of Chicago, Chicago, IL USA; 84 Department of Preventive Medicine, Seoul National University College of Medicine, Seoul, Korea; 85 Department of Biomedical Sciences, Seoul National University Graduate School, Seoul, Korea; 86 Cancer Research Institute, Seoul National University, Seoul, Korea; 87 Department of Genetics and Computational Biology, QIMR Berghofer Medical Research Institute, Brisbane, QLD Australia; 88 Genome Diagnostics Program, IFOM–the FIRC Institute of Molecular Oncology, Milan, Italy; 89 Unit of Molecular Bases of Genetic Risk and Genetic Testing, Department of Research, Fondazione IRCCS Istituto Nazionale dei Tumori (INT), Milan, Italy; 90 Clinical Genetics, Karolinska Institutet, Stockholm, Sweden; 91 Clalit National Cancer Control Center, Carmel Medical Center and Technion Faculty of Medicine, Haifa, Israel; 92 Chronic Disease Epidemiology, Yale School of Public Health, New Haven, CT USA; 93 Center for Molecular Medicine Cologne (CMMC), Faculty of Medicine and University Hospital Cologne, University of Cologne, Cologne, Germany; 94 Department of Internal Medicine, Division of Medical Oncology, University of Kansas Medical Center, Westwood, KS USA; 95 Genomics Center, Centre Hospitalier Universitaire de Québec–Université Laval Research Center, Québec City, QC Canada; 96 Dept of OB/GYN and Comprehensive Cancer Center, Medical University of Vienna, Vienna, Austria; 97 Clinical Genetics Service, Department of Medicine, Memorial Sloan Kettering Cancer Center, New York, NY USA; 98 Department of Tumour Biology, INSERM U830, Paris, France; 99 Université Paris Descartes, Paris, France; 100 Institute of Human Genetics, University Hospital Heidelberg, Heidelberg, Germany; 101 Dept of OB/GYN, Medical University of Vienna, Vienna, Austria; 102 Department of Genetics, Portuguese Oncology Institute, Porto, Portugal; 103 Biomedical Sciences Institute (ICBAS), University of Porto, Porto, Portugal; 104 Breast Cancer Research Programme, Cancer Research Malaysia, Subang Jaya, Selangor Malaysia; 105 Department of Surgery, Faculty of Medicine, University of Malaya, Kuala Lumpur, Malaysia; 106 Department of Clinical Genetics, Odense University Hospital, Odence C, Denmark; 107 Department of Medicine, Magee-Womens Hospital, University of Pittsburgh School of Medicine, Pittsburgh, PA USA; 108 Program in Cancer Genetics, Departments of Human Genetics and Oncology, McGill University, Montréal, QC Canada; 109 Department of Medical Genetics, National Institute for Health Research Cambridge Biomedical Research Centre, University of Cambridge. Vol Box 134, Level 6 Addenbrooke’s Treatment Centre, Addenbrooke’s Hosptital, Cambridge, UK; 110 Department of Cancer Biology and Genetics, The Ohio State University, Columbus, OH USA; 111 Department of Medical Oncology, Beth Israel Deaconess Medical Center, Boston, MA USA; 112 Department of Genetics, University of Pretoria, Arcadia, South Africa; 113 Centro de Investigación en Red de Enfermedades Raras (CIBERER), Madrid, Spain; 114 Fundación Pública Galega de Medicina Xenómica, Santiago de Compostela, Spain; 115 Instituto de Investigación Sanitaria de Santiago de Compostela (IDIS), Complejo Hospitalario Universitario de Santiago, SERGAS, Santiago de Compostela, Spain; 116 Department of Pathology, Leiden University Medical Center, Leiden, The Netherlands; 117 Department of Epidemiology and Biostatistics, Memorial Sloan-Kettering Cancer Center, New York, NY USA; 118 Centre for Cancer Genetic Epidemiology, Department of Oncology, University of Cambridge, Cambridge, UK; 119 Division of Psychosocial Research and Epidemiology, The Netherlands Cancer Institute–Antoni van Leeuwenhoek hospital, Amsterdam, The Netherlands; 120 Peter MacCallum Cancer Center, Melbourne, Victoria Australia; 121 Department of Medicine, St Vincent’s Hospital, The University of Melbourne, Fitzroy, VIC Australia; 122 North East Thames Regional Genetics Service, Great Ormond Street Hospital for Children NHS Trust, London, UK; 123 Leicestershire Clinical Genetics Service, University Hospitals of Leicester NHS Trust, Leicester, UK; 124 North West Thames Regional Genetics Service, Kennedy Galton Centre, The North West London Hospitals NHS Trust, Middlesex, UK; 125 Breast Department, KK Women’s and Children’s Hospital, Singapore, Singapore; 126 Department of Clinical Genetics, Royal Devon & Exeter Hospital, Exeter, UK; 127 Sheffield Clinical Genetics Service, Sheffield Children’s Hospital, Sheffield, UK; 128 Department of Clinical Genetics, South Glasgow University Hospitals, Glasgow, UK; 129 Clinical Genetics Department, St Michael’s Hospital, Bristol, UK; 130 Nottingham Clinical Genetics Service, Nottingham University Hospitals NHS Trust, Nottingham, UK; 131 Faculty of Medicine, University of Southampton, Southampton, UK; 132 North of Scotland Regional Genetics Service, NHS Grampian & University of Aberdeen, Foresterhill, Aberdeen, UK; 133 Southwest Thames Regional Genetics Service, St George’s Hospital, London, UK; 134 Institute of Genetic Medicine, Centre for Life, Newcastle Upon Tyne Hospitals NHS Trust, Newcastle upon Tyne, UK; 135 Department of Clinical Genetics, St George’s, University of London, London, UK; 136 Academic Unit of Clinical and Molecular Oncology, Trinity College Dublin and St James’s Hospital, Dublin, Eire; 137 Department of Population Health Sciences, Weill Cornell Medicine, New York, NY USA; 138 Department of Clinical Genetics, Alder Hey Hospital, Liverpool, UK; 139 Northern Ireland Regional Genetics Centre, Belfast City Hospital, Belfast, UK; 140 West Midlands Regional Genetics Service, Birmingham Women’s Hospital Healthcare NHS Trust, Birmingham, UK; 141 SingHealth Duke-NUS Breast Centre, Singapore, Singapore; 142 South East of Scotland Regional Genetics Service, Western General Hospital, Edinburgh, UK; 143 All Wales Medical Genetics Services, University Hospital of Wales, Cardiff, UK; 144 Princess Anne Hospital, Southampton, UK; 145 Medical Genetics Unit, St George’s, University of London, London, UK; 146 Oxford Regional Genetics Service, Churchill Hospital, Oxford, UK; 147 Oncogénétique, Institut Bergonié, Bordeaux, France; 148 Département de Biopathologie, Centre François Baclesse, Caen, France; 149 Laboratoire d’Oncologie moléculaire, Centre de Lutte Contre le Cancer, Centre Jean Perrin, Clermont-Ferrand, France; 150 Unité de Prévention et d’Epidémiologie Génétique, Centre Léon Bérard, Lyon, France; 151 Département de médicine–Oncogénétique, Gustave Roussy, Villejuif, France; 152 Paris Sciences Lettres Research University, Paris, France; 153 Service de Génétique Biologique, CHRU de Besançon, Besançon, France; 154 Unité d’Oncogénétique, CHU Arnaud de Villeneuve, Montpellier, France; 155 Oncogénétique. Institut de Cancérologie de l’Ouest siteRené Gauducheau, Saint Herblain, France; 156 Unité d’oncogénétique, Centre de Lutte Contre le Cancer, Centre Georges-François Leclerc, Dijon, France; 157 Centre de Génétique. CHU Dijon, Dijon, France; 158 Laboratoire de Génétique Chromosomique, Hôtel Dieu Centre Hospitalier, Chambéry, France; 159 Service Régional Oncogénétique Poitou-Charentes, CH Niort, Niort, France; 160 Service de Génétique, Groupement Hospitalier Est, Hospices Civils de Lyon, Bron, France; 161 Department of Genetics, F76000 and Normandy University, UNIROUEN, Inserm U1245, Normandy Centre for Genomic and Personalized Medicine, Rouen University Hospital, Rouen, France; 162 Département d’Hématologie-Oncologie Médicale, Centre Antoine Lacassagne, Nice, France; 163 Lyon Neuroscience Research Center–CRNL, INSERM U1028, CNRS UMR5292, University of Lyon, Lyon, France; 164 Service de Génétique, Hôpital Bretonneau–CHRU, Tours, France; 165 Service de Génétique Clinique Chromosomique et Moléculaire, Hôpital Nord, CHU Saint Etienne, St Etienne, France; 166 Unité d’Oncogénétique, Centre Paul Strauss, Strasbourg, France; 167 Département Oncologie Génétique, Prévention et Dépistage, Institut Paoli-Calmettes, Marseille, France; 168 Marseille Medical School, Aix-Marseille University, Marseille, France; 169 Laboratoire de génétique médicale, Nancy Université, Centre Hospitalier Régional et Universitaire, Vandoeuvre-les-Nancy, France; 170 Department of Medical Oncology, CHU Dupuytren, Limoges, France; 171 Department of Clinical Genetics, Amsterdam UMC, location AMC, Amsterdam, The Netherlands; 172 Division Laboratories, Pharmacy and Biomedical Genetics, Department of Genetics, University Medical Center Utrecht, Utrecht, The Netherlands; 173 Department of Clinical Genetics, Maastricht University Medical Center, Maastricht, The Netherlands; 174 Department of Surgical Oncology, Family Cancer Clinic, Erasmus MC Cancer Institute, Rotterdam, The Netherlands; 175 Department of Medical Genetics, University Medical Center, Utrecht, The Netherlands; 176 Department of Human Genetics, Radboud University Medical Center, Nijmegen, The Netherlands; 177 Department of Epidemiology, The Netherlands Cancer Institute, Amsterdam, The Netherlands; 178 Department of Genetics, University Medical Center Groningen, University Groningen, Groningen, The Netherlands; 179 Department of Pathology, Erasmus University Medical Center, Rotterdam, The Netherlands; 180 Department of Gynaecology, Family Cancer Clinic, Erasmus MC Cancer Institute, Rotterdam, The Netherlands; 181 Department of Clinical Genetics, VU University Medical Center, Amsterdam, The Netherlands; 182 Clinical Genetics. Amsterdam UMC, Vrije Universiteit Amsterdam, Amsterdam, The Netherlands; 183 Department of Human Genetics and Department of Clinical Genetics, Leiden University Medical Center, Leiden, The Netherlands; 184 Department of Laboratory Medicine and Pathobiology, University of Toronto, Toronto, ON Canada; 185 Laboratory Medicine Program, University Health Network, Toronto, ON Canada

## Abstract

**Purpose:**

To evaluate the association between a previously published 313 variant–based breast cancer (BC) polygenic risk score (PRS_313_) and contralateral breast cancer (CBC) risk, in *BRCA1* and *BRCA2* pathogenic variant heterozygotes.

**Methods:**

We included women of European ancestry with a prevalent first primary invasive BC (*BRCA1* = 6,591 with 1,402 prevalent CBC cases; *BRCA2* = 4,208 with 647 prevalent CBC cases) from the Consortium of Investigators of Modifiers of *BRCA1/2* (CIMBA), a large international retrospective series. Cox regression analysis was performed to assess the association between overall and ER-specific PRS_313_ and CBC risk.

**Results:**

For *BRCA1* heterozygotes the estrogen receptor (ER)-negative PRS_313_ showed the largest association with CBC risk, hazard ratio (HR) per SD = 1.12, 95% confidence interval (CI) (1.06–1.18), C-index = 0.53; for *BRCA2* heterozygotes, this was the ER-positive PRS_313_, HR = 1.15, 95% CI (1.07–1.25), C-index = 0.57. Adjusting for family history, age at diagnosis, treatment, or pathological characteristics for the first BC did not change association effect sizes. For women developing first BC < age 40 years, the cumulative PRS_313_ 5th and 95th percentile 10-year CBC risks were 22% and 32% for *BRCA1* and 13% and 23% for *BRCA2* heterozygotes, respectively.

**Conclusion:**

The PRS_313_ can be used to refine individual CBC risks for *BRCA1/2* heterozygotes of European ancestry, however the PRS_313_ needs to be considered in the context of a multifactorial risk model to evaluate whether it might influence clinical decision-making.

## INTRODUCTION

Heterozygotes of germline pathogenic variants in *BRCA1* or *BRCA2* (henceforth *BRCA1/2* heterozygotes) have a higher risk of developing contralateral breast cancer than nonheterozygotes.^1^ The estimated cumulative 10-year contralateral breast cancer risk varies across studies between 18.5% and 34.2% for *BRCA1* heterozygotes and between 10.8% and 29.2% for *BRCA2* heterozygotes,^1–6^ compared to 4–6% in the population.^7,8^ Whether or not to undergo a risk-reducing contralateral mastectomy, which is an invasive intervention and associated with side effects such as postoperative surgical complications, inability to breast feed in the future, and psychosocial burden,^9^ is an important and difficult decision for *BRCA1/2* heterozygotes who have been just confronted with their first breast cancer diagnosis. Precise individualized risk estimates could facilitate decision making for these women.

Two important factors influencing contralateral breast cancer risk in *BRCA1/2* heterozygotes are the age at diagnosis of the first breast tumor and a family history of breast cancer.^2,4,5,10^ The effect of family history on contralateral breast cancer risk suggests a role for other genetic factors. In the last decade, more than 180 common low risk variants have been associated with breast cancer risk in genome-wide association studies (GWAS).^11–13^ Individually, these variants are associated with small increases in risk, but when combined as polygenic risk scores (PRS) they may improve disease-related risk stratification for women of European and Asian ancestry in the population.^14–16^ A limited number of studies have shown that variants associated with the risk of a first primary breast cancer are also associated with the risk of contralateral breast cancer.^17–19^ Furthermore, the PRS derived from the general population has also been shown to be associated with breast cancer risk in *BRCA1/2* heterozygotes.^20–24^

The most predictive, well validated PRS for breast cancer in the general population is based on 313 breast cancer–associated variants (PRS_313_); it showed an association with breast cancer in ten prospective studies with an odds ratio (OR) per standard deviation (SD) of 1.61 and an area under the receiver–operator characteristic curve of 0.630.^14^ Among *BRCA2* heterozygotes, this same PRS_313_ was also associated with breast cancer risk, hazard ratio (HR) per SD = 1.31, 95% confidence interval (CI) (1.27–1.36).^24^ Among *BRCA1* heterozygotes, the largest association with breast cancer risk was found using the estrogen receptor (ER)-negative PRS_313_ (which uses the same variants but with weights adapted to provide better prediction for ER-negative disease), HR = 1.29, 95% CI (1.25–1.33).^24^ Although these effect sizes were smaller than those for the general population, the 313 variant–based PRS could have a substantial impact on the high absolute risks^24^ associated with *BRCA1/2* pathogenic variants.^25^ Whether variants associated with breast cancer are associated with contralateral breast cancer risk for *BRCA1/2* heterozygotes as well, individually or combined in a PRS, has not been investigated previously. If so, the PRS may be useful to guide choices for risk management, especially regarding invasive risk-reducing contralateral mastectomy. In this study, we investigated whether the 313 variant–based PRS for breast cancer is associated with contralateral breast cancer risk among women of European ancestry with pathogenic variants in *BRCA1/2* and explored the implications for contralateral breast cancer risk prediction for these women.

## MATERIALS AND METHODS

### Study participants

We used retrospective cohort data from heterozygotes participating in the Consortium of Investigators of Modifiers of *BRCA1/2* (CIMBA).^26^ Briefly, CIMBA participants are heterozygotes of pathogenic variants in *BRCA1* or *BRCA2* who are 18 years or older at the time of inclusion and have phenotypic data available.^26^ CIMBA includes 81 individual studies of which the majority of the participants were ascertained through cancer genetics clinics.^26^ Although studies in CIMBA include individuals of non-European ancestry, our analyses were, due to power considerations (small numbers available for analyses and expected lower estimates for the PRS_313_ in Asian ancestry based on results of women in the general breast cancer population^19^), restricted to women of European ancestry with available array genotyping data (31,195 women of 67 studies).

Women were eligible for this retrospective analysis if they developed an invasive primary breast tumor without metastatic disease at least 1 year before the baseline age. Women without information about metastatic disease were assumed to have no metastatic disease (*n* = 9,242 of whom 2,140 had a known negative lymph node status). Baseline age was defined as the age at local ascertainment (97%), or when this was not known, age at genetic testing (2%) or age at last follow-up (1%). Women were excluded if no information was available about the age at baseline or if they had developed synchronous contralateral breast cancer. Synchronous contralateral breast cancer was defined as contralateral breast cancer within one year after the first primary breast cancer, which was based on the exact date of cancer diagnosis or, if this was not available, on the age at diagnosis. A schematic overview of the selection is shown in Fig. S1. In total, 6,591 women with *BRCA1* and 4,208 women with *BRCA2* pathogenic variants were included in this study, among whom 1,402 *BRCA1* heterozygotes and 647 *BRCA2* heterozygotes have had contralateral breast cancer. The diagnosis of primary and contralateral breast cancer was confirmed by pathology records, tumor registry data, or medical records by the individual studies. Available phenotypic information for all participants is shown in Table 1, including the number of participants for whom the information was not available for each of the variables. Information about the ER status of the first primary breast cancer compared to the contralateral breast cancer is shown in Table S1.

**Table 1:** Characteristics of the participants.

		*BRCA1* heterozygotes		*BRCA2* heterozygotes	
		UBC, *n* (%)	CBC, *n* (%)	UBC, *n* (%)	CBC, *n* (%)
*N*		5,189	1,402	3,561	647
Genotyping array	iCOGS	895 (17)	200 (14)	383 (11)	80 (12)
	OncoArray	4,294 (83)	1,202 (86)	3,178 (89)	567 (88)
Birth cohort	<1920	25 (0.5)	8 (0.6)	23 (0.6)	9 (1)
	1920–1929	143 (3)	46 (3)	121 (3)	30 (5)
	1930–1939	392 (8)	130 (9)	341 (10)	99 (15)
	1940–1949	1,060 (20)	386 (28)	793 (22)	172 (27)
	1950–1959	1,540 (30)	452 (32)	1,104 (31)	202 (31)
	1960–1969	1,354 (26)	298 (21)	822 (23)	115 (18)
	≥1970	675 (13)	82 (6)	357 (10)	20 (3)
Variant class^a^	I	3,354 (65)	904 (64)	3,207 (90)	570 (88)
	II	1,345 (26)	374 (27)	125 (4)	25 (4)
	III	490 (9)	124 (9)	229 (6)	52 (8)
BRRM		160 (3)	0	101 (3)	0
Deceased	*N*	44 (0.8)	12 (0.9)	19 (0.5)	2 (0.3)
Family history^b^	No BC	583 (11)	175 (12)	289 (8)	78 (12)
	1 BC	906 (17)	270 (19)	760 (21)	127 (20)
	≥ 2 BC	1,250 (24)	363 (26)	1,120 (31)	210 (32)
	Unknown	2,450 (47)	594 (42)	1,392 (39)	232 (36)
**Characteristics of first BC**					
Age at diagnosis	Mean	41.8	38.5	44.5	41.8
	Range	19–82	19–68	18–85	21–75
ER status	Positive	570 (11)	92 (7)	1,302 (37)	182 (28)
	Negative	1,738 (33)	402 (29)	424 (12)	61 (9)
	Unknown	2,881 (56)	908 (65)	1,835 (52)	404 (62)
Node status	Positive	797 (15)	182 (13)	781 (22)	119 (18)
	Negative	1,544 (30)	441 (31)	877 (25)	151 (23)
	Unknown	2,848 (55)	779 56)	1,903 (53)	377 (58)
Tumor size^c^	T1	1,261 (24)	314 (22)	842 (24)	136 (21)
	T2	771 (15)	211 (15)	553 (16)	87 (13)
	T3	67 (13)	12 (0.9)	78 (2)	8 (1)
	T4	16 (0.5)	2 (0.1)	22 (0.6)	2 (0.3)
	Unknown	3,074 (59)	863 (62)	2,066 (58)	414 (64)
Chemotherapy^d^	Yes	1,099 (21)	236 (17)	821 (23)	123 (19)
	No	576 (11)	212 (15)	503 (14)	129 (20)
	Unknown	3,514 (68)	954 (68)	2,237 (63)	395 (61)
Adjuvant hormone therapy	Yes	493 (10)	125 (9)	795 (22)	111 (17)
	No	1,103 (21)	288 (21)	474 (13)	135 (21)
	Unknown	3,593 (69)	989 (71)	2,292 (64)	401 (62)
Adjuvant trastuzumab therapy	Yes	11 (0.2)	1 (0.1)	20 (0.6)	0 (0)
	No	1,161 (22)	351 (25)	983 (28)	218 (34)
	Unknown	4,017 (77)	1,050 (75)	2,558 (72)	429 (66)
Radiotherapy	Yes	1,090 (21)	277 (20)	797 (22)	158 (24)
	No	535 (10)	141 (10)	420 (12)	84 (13)
	Unknown	3,564 (69)	984 (70)	2,344 (66)	405 (63)
**Characteristics of CBC**					
Age at diagnosis	Mean	–	47.3	–	51.24
	Range	–	26–80.5	–	23.8–86
Invasiveness	Invasive	–	1,267 (90)	–	545 (84)
	Noninvasive	–	135 (10)	–	102 (16)
ER status	Positive	–	101 (7)	–	197 (30)
	Negative	–	446 (32)	–	50 (8)
	Unknown	–	855 (61)	–	400 (62)
**PRS**_**313**_					
Standardized PRS_313_ mean (SD)	Overall BC	0.08 (1.01)	0.13 (1.01)	0.09 (1.02)	0.27 (1.04)
	ER-positive BC	0.07 (1.01)	0.09 (1.01)	0.08 (1.01)	0.27 (1.03)
	ER-negative BC	0.09 (1.00)	0.23 (0.99)	0.07 (1.02)	0.23 (1.07)

*BC* breast cancer, *BRRM* bilateral risk-reducing mastectomy, *CBC* contralateral breast cancer, *ER* status estrogen receptor status of the tumor, *PRS* polygenic risk score, *SD* standard deviation, *UBC* unilateral breast cancer.

^a^Variant class: I = unstable or no protein, II = stable mutant protein, III = consequence unknown.

^b^Family history was defined as the number of first- or second-degree relatives affected with BC, ranging from 0 to ≥2.

^c^Tumor size: T1 = ≤ 2 cm (≤0.79 inches), T2 = > 2cm-5cm (>0.79–1.97 inches), T3 = > 5 cm (>1.97 inches), T4 = any size, with direct extension to the chest wall or skin.

^d^Including neoadjuvant and adjuvant chemotherapy.

### Genotyping and polygenic risk score calculation

For most of the participants, genotyping was performed with the Illumina OncoArray.^27^ The remaining participants were genotyped with the Illumina iCOGS array.^11^ Details about the quality control procedures and correlation between the arrays have been described previously.^19,24,28–31^ European ancestry was determined using genetic data and multidimensional scaling. More detailed information about the genotyping and PRS calculation is provided in the Supplementary methods.

We used the 313 variant–based PRS for breast cancer developed in an independent study using data from the general population as described previously;^14^ correlation between PRS based on the two genotyping arrays was high.^19^ The PRS for overall breast cancer (PRS_313_) and two ER-specific PRS, the ER-positive PRS_313_ and ER-negative PRS_313_ were calculated. The variants and their corresponding weights used in the PRS as published previously^14^ and the imputation quality are listed in Table S2. The three PRS were standardized to the mean from all CIMBA participants, including both unaffected and affected women, and to the SD in Breast Cancer Association Consortium (BCAC) population controls that were included in the validation data set.^14^ Using these SDs, the HR estimates for the associations of the standardized PRS_313_ in our study are directly comparable with the OR estimates reported in the BCAC population-based study^14^ and the HR estimates reported for primary breast cancer in *BRCA1* and *BRCA2* heterozygotes.^24^

### Statistical analysis

To assess the associations between the three PRS and contralateral breast cancer risk in *BRCA1/2* heterozygotes, Cox regression analyses were performed. The time at risk was started one year after the first breast cancer diagnosis based on the exact date, or if not available, on the age of developing the first breast tumor. Time at risk of participants was censored at age at baseline, i.e., end of follow-up in these analyses, prophylactic contralateral mastectomy, or death, whichever was earlier (Fig. S2). Incidence of a metachronous contralateral breast cancer, invasive or in situ, before baseline was considered as an event in the main analyses. The proportional hazard assumption was evaluated by using Schoenfeld residuals against the transformed time. A sensitivity analysis was performed considering invasive contralateral breast cancer only as an event. Women who developed an in situ contralateral breast cancer were censored at the age at diagnosis of the in situ contralateral breast cancer. Furthermore, a sensitivity analysis was performed including information about distant relapse, which was available for 1,725 *BRCA1* and 1,450 *BRCA2* heterozygotes. In total 55 *BRCA1* heterozygotes and 101 *BRCA2* heterozygotes were censored at the age of distant relapse of which 13 and 11 women were excluded from the analyses, respectively, because they developed distant relapse in the year before the baseline age.

Analyses were stratified by country (Table S3), adjusted for birth cohort (quartiles of the observed distribution), and clustered on family membership using a unique family identifier to account for the inclusion of related individuals. For *BRCA1* and *BRCA2* respectively, there were 5,923 and 3,752 clusters, of which 554 and 362 clusters had more than one participant. The main analyses assessed the association with the PRS as a continuous covariate. We evaluated the linearity of the association using restricted cubic splines with three knots, which showed no evidence for violation of the linearity assumption. The discriminatory ability of the best-performing PRS was evaluated by Harrell’s C-index.^32^ C-indexes were calculated stratified by country and clustered on family membership.

The influence of possible confounding variables on the observed associations was assessed using the PRS exhibiting the largest associations. Possible confounding variables included breast cancer family history, age at diagnosis of the first breast cancer, pathological characteristics, and treatment of the first breast cancer. Each variable was added to the model one by one and in addition, a full model that included all possible confounders together was fitted. If the addition of a variable resulted in a change of more than 10% in the log HR, the variable was retained as a covariate in the final Cox regression model. To avoid excluding many participants with missing data for one of these included variables (Table 1), missing data were imputed using multiple imputation by chained equations (MICE).^33^ Imputation was started with the least missing variable and progressed in order of increased amount of missing data. Using this method, ten complete data sets for analyses were created and mean parameter estimates were derived.

Secondary analyses were performed for ER-positive and ER-negative cases only, based on the ER status of the contralateral breast cancer, after imputation as described above. The average number of ER-positive and ER-negative cases in the ten imputed data sets is shown in Table S4. In these analyses the event of interest was either ER-positive or ER-negative contralateral breast cancer. Contralateral breast cancer cases with the alternative ER status were censored at the age of contralateral breast cancer.

The interaction between the PRS with the age at first breast cancer diagnosis was tested in the final model, treating the PRS as a continuous variable. Furthermore, the effect size of the PRS was evaluated for groups based on the age at first primary breast cancer diagnosis (<40 years; 40 to 50 years; ≥50 years).^1,20^ The association of the PRS and contralateral breast cancer risk was tested separately for heterozygotes of pathogenic variants that lead to unstable or no protein (class I) and heterozygotes of pathogenic variants that lead to mutant stable protein (class II). Finally, analyses were performed to test the association between a categorized PRS and contralateral breast cancer risk to establish whether the results were consistent with those under a continuous PRS model. The categories were defined on the basis of the distribution of the PRS in unilateral breast cancer cases, using PRS percentiles (0–5th, 5th−10th, 10th−20th, 20th−40th, 40th−60th [reference], 60th−80th, 80th−90th, 90th−95th, 95th−100th).

### Cumulative risks

Absolute contralateral breast cancer risks were calculated at percentiles of the best-performing continuous PRS for both *BRCA1* and *BRCA2* heterozygotes, using the log HR per SD and including an interaction term with the continuous age at first breast cancer diagnosis (at age 35, 45, and 55 for the corresponding age groups as described below). For this purpose, we constrained the incidence of contralateral breast cancer, by age at first breast cancer and in years after the first breast cancer, and averaged over all PRS categories to agree with external contralateral breast cancer incidence estimates, as described previously.^23^ These external incidence estimates were based on prospective cohort data from three consortia on heterozygotes of pathogenic *BRCA1* and *BRCA2* variants,^1^ the International *BRCA1/2* Carrier Cohort Study (IBCCS), the Breast Cancer Family Registry (BCFR), and the Kathleen Cunningham Foundation Consortium for Research Into Familial Breast Cancer (kConFab). Because the contralateral breast cancer incidences vary with the age of first breast cancer diagnosis, incidences were calculated for three different groups based on the age of the first breast cancer diagnosis (<40 years, 40 to 50 years, ≥50 years).^1^

All statistical tests were performed with R version 3.5.0.^34^ Statistical significance was defined as a two-sided *p* value <0.05.

## RESULTS

In the analyses, 6,591 *BRCA1* and 4,208 *BRCA2* heterozygotes of European ancestry who had developed an invasive first primary breast cancer before entry in CIMBA were identified. The median follow-up time was 6.0 and 5.4 years for *BRCA1* and *BRCA2* heterozygotes, respectively. In total, 1,402 *BRCA1* and 647 *BRCA2* heterozygotes were diagnosed with a metachronous contralateral breast cancer before enrollment in CIMBA. The cumulative 10-year risk of developing contralateral breast cancer in this cohort was 25%, 95% CI (23.5–26.4%) and 18.8%, 95% CI (17.1–20.5%) for *BRCA1* and *BRCA2* heterozygotes, respectively (Fig. S3). Patient and tumor characteristics as well as the PRS distributions are shown in Table 1 and Fig. S4.

### PRS and contralateral breast cancer risk

**Fig. 1:**
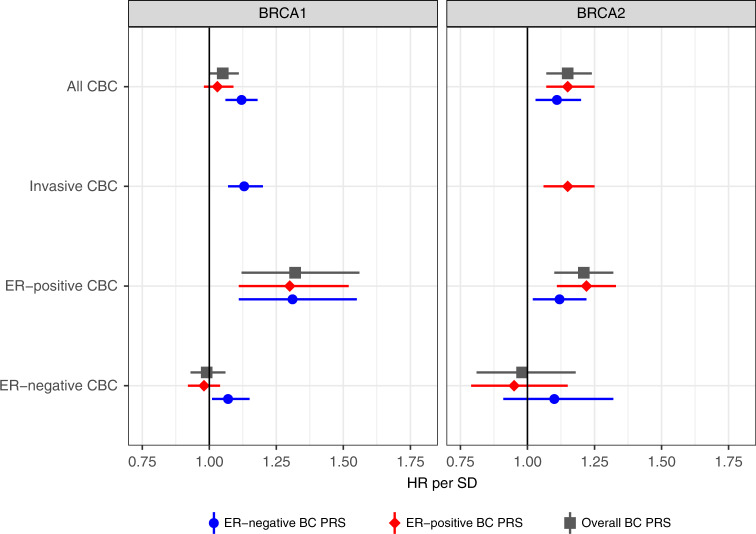
Association between the PRS and contralateral breast cancer risk for *BRCA1* and *BRCA2* heterozygotes. Effect size of the association between contralateral breast cancer and the three different PRS_313_ after testing for covariates for the following selections: all contralateral breast cancer, invasive contralateral breast cancer only, ER-negative contralateral breast cancer, and ER-positive contralateral breast cancer. The numbers of unilateral and contralateral breast cancer cases and effect sizes are shown in Table 2 and Table S4. CBC contralateral breast cancer, ER estrogen receptor, HR hazard ratio, PRS polygenic risk score, SD standard deviation.

Results of the association analyses between the PRS and contralateral breast cancer risk are shown in Table 2, Table S4, and Fig. 1.

**Table 2:** Results of association analyses between the PRS_313_ and contralateral breast cancer risk.

		*BRCA1* heterozygotes (ER-negative PRS_313_)					*BRCA2* heterozygotes (ER-positive PRS_313_)				
		UBC cases, *n*	CBC cases, *n*	HR^a^	95% CI	*P*	UBC cases, *n*	CBC cases, *n*	HR^a^	95% CI	*P*
PRS continuous	All CBC	5,189	1,402	1.12	1.06–1.18	5.98×10^-5^	3,561	647	1.15	1.07–1.25	1.94×10^-4^
	Invasive CBC	5,324	1,267	1.13	1.07–1.20	3.15×10^-5^	3,663	545	1.15	1.06–1.25	6.02×10^-4^
Categorical PRS percentiles	0–5	260	48	0.81	0.59–1.11	0.188	166	28	1.06	0.71–1.58	0.782
	5–10	259	54	0.77	0.57–1.03	0.082	198	26	0.68	0.44–1.04	0.074
	10–20	519	131	0.94	0.76–1.15	0.544	355	51	0.91	0.66–1.25	0.554
	20–40	1,038	230	0.83	0.70–0.98	0.031	697	108	0.87	0.68–1.13	0.295
	40–60 (reference)	1,037	282	1.00			695	123	1.00		
	60–80	1,038	313	1.04	0.88–1.22	0.664	734	128	0.96	0.75–1.23	0.748
	80–90	519	170	1.11	0.92–1.34	0.255	358	90	1.35	1.03–1.77	0.030
	90–95	259	82	1.18	0.92–1.51	0.185	178	46	1.35	0.96–1.90	0.082
	95–100	260	92	1.24	0.98–1.56	0.074	180	47	1.31	0.94–1.82	0.116
PRS*age BC1 continuous	Main effect	5,189	1,402	1.48	1.15–1.89	2.03×10^-3^	3,561	647	1.53	1.11–2.12	0.010
	Interaction effect			0.99	0.99–1.00	0.025			0.99	0.99–1.00	0.089
PRS effect per age group	<40	2,339	815	1.22	1.14–1.31	4.79×10^-8^	1,238	268	1.23	1.09–1.38	5.78×10^-4^
	40–50	1,821	456	0.99	0.90–1.09	0.785	1,306	261	1.19	1.05–1.34	6.91×10^-3^
	≥50	1,029	131	1.03	0.86–1.24	0.715	1,017	118	0.97	0.81–1.15	0.698
Variant class^b^	Class I	3,354	904	1.11	1.03–1.18	4.32×10^-3^	3,207	570	1.16	1.07–1.26	1.99×10^-4^
	Class II	1,345	374	1.15	1.04–1.28	4.75×10^-3^	125	25	0.91	0.65–1.28	0.594

*BC1* first primary breast cancer, *CBC* contralateral breast cancer, *CI* confidence interval, *HR* hazard ratio, *PRS* polygenic risk score, *UBC* unilateral breast cancer.

^a^HRs for association with breast cancer and the continuous PRS_313_ are reported per standard deviation of the PRS in population-based controls.

^b^Class I pathogenic variants result in an unstable or no protein. Class II pathogenic variants yield stable mutant proteins.

### *BRCA1* heterozygotes

For *BRCA1* heterozygotes the ER-negative PRS_313_ showed the largest association with all contralateral breast cancer, HR per SD = 1.12, 95% CI (1.06–1.18), *p* value = 6.0×10^−5^, C-index 0.53, 95% CI (0.51–0.55). There was no evidence of violation of the proportional hazard assumption, *p* value = 0.840.

Neither sequential inclusion of possible confounders nor including all these confounders in one model changed the log HR estimate for the ER-negative PRS_313_ association more than 10% when compared with the model with no confounders (Table S5).

Considering only invasive contralateral breast cancer as the event of interest resulted in a similar association with the ER-negative PRS_313_, HR per SD = 1.13, 95% CI (1.07–1.20), *p* value = 3.2×10^−5^.

Censoring at distant metastasis relapse, if applicable, did not change the effect size of the ER-negative PRS_313_, HR per SD = 1.12, 95% CI (1.06–1.18), *p* value = 4.9×10^-5^.

**Fig. 2:**
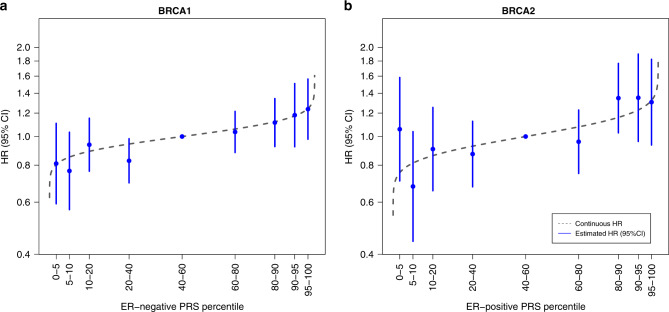
Association between categories of the PRS and contralateral breast cancer risk for *BRCA1* and *BRCA2* heterozygotes. HRs and 95% CI for percentiles of the ER-negative PRS_313_ for *BRCA1* heterozygotes and the ER-positive PRS_313_ for *BRCA2* heterozygotes, relative to the middle quintile. The PRS percentile groups were 0–5%, 5–10%, 10–20%, 20–40%, 40–60% (reference), 60–80%, 80–90%, 90–95%, and 95–100% based on the distribution in unilateral breast cancer cases. The numbers and corresponding effect sizes are shown in Table 2. The gray line represents the distribution based on the HR of the continuous ER-negative PRS_313_ and ER-positive PRS_313_ and the distribution in unilateral breast cancer cases of *BRCA1* and *BRCA2* heterozygotes respectively. CI confidence interval, ER estrogen receptor, HR hazard ratio, PRS polygenic risk score.

The HR estimates for association with contralateral breast cancer for different quantiles of the ER-negative PRS_313_, were consistent with the predicted HRs from the model using the continuous ER-negative PRS_313_ (Table 2 and Fig. 2).

For ER-positive contralateral breast cancer as event, the PRS_313_ showed the largest association, HR per SD = 1.32, 95% CI (1.12–1.56), *p* value = 0.002. For ER-negative contralateral breast cancer as event, only the ER-negative PRS_313_ showed a significant association, HR per SD = 1.07, 95% CI (1.01–1.15), *p* value = 0.036 (Table S4).

### *BRCA2* heterozygotes

For *BRCA2* heterozygotes the largest association was seen with the ER-positive PRS_313_, HR per SD = 1.15, 95% CI (1.07–1.25), *p* value = 1.9×10^−4^, C-index 0.57, 95% CI (0.54–0.59). There was no evidence of violation of the proportional hazard assumption, *p* value = 0.300.

Neither sequential inclusion of possible confounders, nor including all these confounders in one model, changed the log HR estimate for the ER-positive PRS_313_ association more than 10% when compared with the model with no confounders (Table S5).

Considering only invasive contralateral breast cancer as the event of interest resulted in a similar association, HR per SD for the ER-positive PRS_313_ = 1.15, 95% CI (1.06–1.25), *p* value = 6.0×10^−4^.

Censoring at distant metastasis relapse, if applicable, did not change the effect size of the ER-positive PRS_313_, HR per SD = 1.15, 95% CI (1.07–1.24), *p* value = 2.1×10^-4^.

The HR estimates for association with contralateral breast cancer for different quantiles of the ER-positive PRS_313_, were consistent with the predicted estimates using the continuous PRS_313_ (Table 2 and Fig. 2).

The ER-positive PRS_313_ showed the largest association with ER-positive contralateral breast cancer for *BRCA2* heterozygotes, HR per SD = 1.22, 95% CI (1.11–1.33), *p* value = 2.2×10^−5^ (Table S4). None of the PRS showed significant associations with ER-negative contralateral breast cancer for *BRCA2* heterozygotes, but the ER-negative PRS_313_ exhibited the largest HR estimate, HR per SD = 1.10, 95% CI (0.91–1.32), *p* value = 0.346.

### Interaction with age at first breast cancer diagnosis

A significant interaction between the age at first breast cancer diagnosis and the ER-negative PRS_313_ was found for *BRCA1* heterozygotes: HR per year = 0.99, 95% CI (0.99–1.00), *p* value = 0.025. For *BRCA2* heterozygotes a similar magnitude of interaction was observed with the ER-positive PRS_313_, although the interaction was not significant, HR per year = 0.99, 95% CI (0.99–1.00), *p* value = 0.09.

Categorizing age at first breast cancer diagnosis for *BRCA1* heterozygotes resulted in HRs per SD of the ER-negative PRS_313_ of 1.22, 95% CI (1.14–1.31); 0.99, 95% CI (0.90–1.09);, and 1.03, 95% CI (0.86–1.24) for ages <40 years, 40–50 years, and ≥50 year respectively. For *BRCA2* heterozygotes the corresponding estimates for ER-positive PRS_313_ were 1.23, 95% CI (1.09–1.38); 1.19, 95% CI (1.05–1.34); and 0.97, 95% CI (0.81–1.15) respectively (Table 2).

### Analyses by predicted variant effect on protein expression

For *BRCA1* heterozygotes, the HRs for association between the ER-negative PRS_313_ and contralateral breast cancer risk were similar for heterozygotes of pathogenic variants, which lead to a stable mutant protein (class II) compared with those leading to no protein or an unstable protein (class I). For *BRCA2* heterozygotes, the ER-positive PRS_313_ effect size for the association with contralateral breast cancer risk was nonsignificantly smaller among heterozygotes of a pathogenic variant that lead to a stable mutant protein, although statistical power to detect these associations was low and the confidence intervals overlap with the overall estimate (Table 2).

### Cumulative risks

**Fig. 3:**
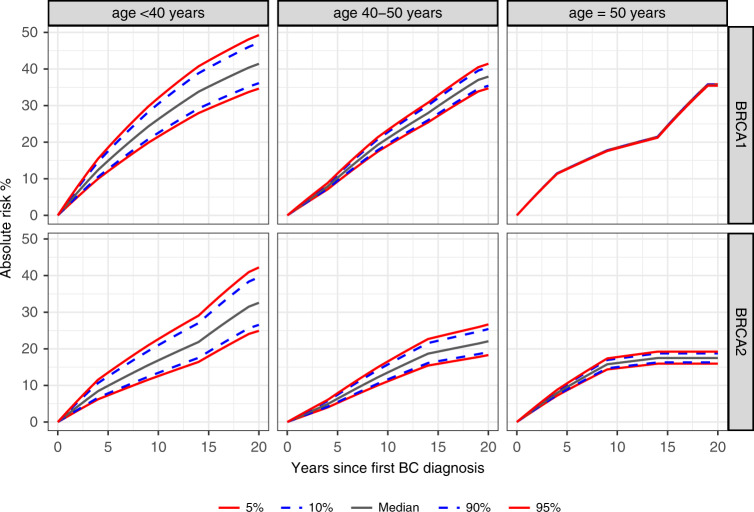
Absolute contralateral breast cancer risk by PRS percentiles per age category of the first breast cancer diagnosis for *BRCA1* and *BRCA2* heterozygotes. Predicted absolute contralateral breast cancer risks by percentile of the continuous ER-negative PRS_313_ for *BRCA1* heterozygotes and ER-positive PRS_313_ for *BRCA2* heterozygotes. The assumed contralateral breast cancer incidences were from a study that estimated breast cancer incidence in a large prospective cohort of *BRCA1* and *BRCA2* heterozygotes.^20^ The age categories were based on the age at diagnosis of the first primary breast tumor. Risks were calculated including the interaction between the PRS and the continuous age of first breast cancer diagnosis. The lines for different percentiles of the PRS are overlapping for the age category ≥50 year for *BRCA1* heterozygotes. BC breast cancer, CBC contralateral breast cancer, PRS polygenic risk score.

Estimate cumulative contralateral breast cancer risks, by categories of age at diagnosis of the first breast cancer are shown in Fig. 3. The largest risk difference was seen for women with a first breast cancer diagnosis before the age of 40, with *BRCA1* heterozygotes at the 5th percentile of the ER-negative PRS_313_ having a 10- and 20-year risk of 22% and 35% compared with 32% and 49% at the 95th percentile, respectively. For *BRCA2* heterozygotes, the 10- and 20-year risks in this category were 13% and 25% at the 5th percentile of the ER-positive PRS_313_ compared with 23% and 42% for women at the 95th percentile.

## DISCUSSION

In this study we investigated the associations between an established PRS based on 313 variants for primary first breast cancer and contralateral breast cancer risks among *BRCA1* and *BRCA2* heterozygotes of European ancestry enrolled in the large international retrospective CIMBA cohort. We showed significant albeit modest associations among both *BRCA1* and *BRCA2* heterozygotes between the PRS and contralateral breast cancer risk. For *BRCA1* heterozygotes, the largest association was seen with the ER-negative PRS_313_, while for *BRCA2* heterozygotes, both the PRS_313_ and ER-positive PRS_313_ showed similar associations with contralateral breast cancer risk that were somewhat larger than the ER-negative PRS_313_ association. These findings are consistent with previous studies on the effects of disease-specific PRS on the first breast cancers in *BRCA1 and BRCA2* heterozygotes^20,24^ and with the higher relative prevalence of ER-negative and ER-positive contralateral breast cancers respectively, in this cohort.

For both *BRCA1* and *BRCA2* heterozygotes, the strength of the association was greater for ER-positive contralateral breast cancers compared with ER-negative contralateral breast cancers (in the case of *BRCA1*, even if the ER-negative PRS was used), although most of the confidence intervals overlapped. The effect sizes for the PRS are also larger for ER-positive disease in the general population, perhaps because ER-positive disease is commoner and the power to identify genetic variants has been greater for ER-positive disease. With larger data sets, it should be possible to develop better subtype specific PRS for contralateral breast cancer.

Although we found clear associations between the PRS and contralateral breast cancer risk, the magnitude of these associations (expressed in terms of HRs) were smaller than previously reported for the first breast cancers. For *BRCA1* heterozygotes, the HR per SD for the association between the ER-negative PRS_313_ and breast cancer was 1.29, 95% CI (1.25–1.33),^24^ compared with 1.12, 95% CI (1.06–1.18) for contralateral breast cancer in this study. For *BRCA2* heterozygotes, the HR per SD for the association between the ER-positive PRS_313_ and breast cancer was 1.31, 95% CI (1.26–1.36),^24^ compared with 1.15, 95% CI (1.07–1.24) for contralateral breast cancer in this study. This lower relative risk is consistent with a general pattern of a lower relative risk in a higher risk population, as seen in the lower relative risk for contralateral breast cancer than first breast cancer in the general population,^19^ and the lower relative risk for the first cancer in *BRCA1/2* heterozygotes than in the general population.^24^ The attenuated estimate might be explained by several factors, some of which are speculative. *BRCA1/2* pathogenic variant heterozygotes in this study were selected based on having a first breast cancer; these women will have on average a higher PRS, but also higher frequencies of other genetic and nongenetic risk factors than women who do not develop breast cancer at all. This can lead to a weaker association with the PRS as women with the largest PRS may have lower risks due to other factors, a phenomenon related to index event bias.^35^ There could also be negative interactions between the PRS effect and other risk factors (for example, treatment factors). However, in this study, we have shown that adjustment for the known contralateral breast cancer risk factors did not change the effect size of the PRS, which was also shown in population-based studies.^17,19^ Finally, although we tried to exclude potential early metastases misdiagnosed as second primaries by excluding women who developed a contralateral breast cancer the first year after the primary diagnosis, it is possible that a small percentage of contralateral breast cancers were metastases.^36^

A limitation of this study is that participants were recruited through clinical genetic centers, resulting in ascertainment bias, as individuals are more likely to have a strong family of breast cancer and/or be affected at a young age to be referred for testing. This was a historical cohort in which follow-up was prior to entry into CIMBA, so that all cases are prevalent. Therefore, the breast cancer patients included in the analyses are likely to be at higher contralateral breast cancer risk when compared with the general *BRCA1/2* heterozygote breast cancer population. Indeed, the estimated 20-year risks of developing contralateral breast cancer in this study were higher compared to a previously published study with a prospective design:^1^ 47% versus 40% for *BRCA1* heterozygotes and 40% versus 26% for *BRCA2* heterozygotes, respectively. While this is unlikely to introduce a significant bias in the relative risk estimates, a prospective cohort would clearly be preferably, although this will take several years to achieve. Finally, the PRS was developed using data sets of women of European ancestry, since our data set included insufficient samples of women of other ancestries, and our results were exclusively based on women of European ancestry. Therefore, caution is required when applying this to non-European ancestry populations. However, a population study found clear associations between the PRS, based on the same 313 variants or a subset of these variants, and (contralateral) breast cancer also in women of Asian ancestry. The effect size of these associations were slightly weaker, possibly reflecting the fact that this PRS was developed in a cohort of women of European ancestry.^16,19^ These results suggest that there might be an association with the PRS as well in *BRCA1/2* heterozygotes of Asian ancestry. Future studies including a sufficient number of individuals of Asian ancestry are needed to confirm this statement.

Although the relative risks of the PRS for contralateral breast cancer were modest, differences in the PRS may still have an important effect on the absolute risk, which is high. *BRCA1* and *BRCA2* heterozygotes under age 40 at first breast cancer, at the 5th and 95th percentile of the PRS, differed by 10% in 10-year contralateral breast cancer risk. These absolute risk differences are modest, but might be of relevance for the choices regarding preventive surgery if incorporated into a multifactorial model that includes other predictive factors, such as family history and adjuvant systemic treatment of the first breast cancer.^37,38^ In the context of such a comprehensive model, further research is needed to investigate whether the PRS would contribute to the choices that women make for follow-up or preventive surgery.

To summarize, we have investigated the associations between PRS based on 313 variants with contralateral breast cancer risk in a large international series of *BRCA1/2* heterozygotes. We found that the PRS is associated with contralateral breast cancer risk in both *BRCA1* and *BRCA2* heterozygotes of European ancestry and that PRS can be used to refine estimates of contralateral breast cancer risks in these women. However, for women with a first breast cancer after the age of 50, PRS may be of less value in the prediction of the contralateral breast cancer risk. Incorporating risk factors other than PRS and including ER-specific estimates may further improve contralateral breast cancer risk prediction. Before implementation in a diagnostic setting, our results should be validated in a prospective cohort of *BRCA1* and *BRCA2* heterozygotes.

## Supplementary information

Supplementary information

Supplementary table S2

## Data Availability

CIMBA data is available on request. To receive access to the data, a concept form must be submitted, which will then be reviewed by the CIMBA Data Access Coordination Committee (DACC). Please contact Lesley McGuffog (email: ljm26@medschl.cam.ac.uk) to get access to these concept forms (http://cimba.ccge.medschl.cam.ac.uk/contact/).
