# Supplementary material for: The predictive ability of the 313 variant–based polygenic risk score for contralateral breast cancer risk prediction in women of European ancestry with a heterozygous BRCA1 or BRCA2 pathogenic variant
Source: Genet Med. 2021 Jun 10;23(9):1726–37. doi: 10.1038/s41436-021-01198-7 (PMC8460445; doi:10.1038/s41436-021-01198-7)
Supplement: Supplementary file 1 — Supplementary information [file 41436_2021_1198_MOESM1_ESM.pdf]

# Supplementary information

Lakeman et al. The predictive ability of the 313-variant-based polygenic risk score for contralateral breast cancer risk prediction in women of European ancestry with a heterozygote *BRCA1* or *BRCA2* pathogenic variant

## Supplementary methods

### Genotyping and Polygenic Risk Score calculation

For most of the participants, genotyping was performed with the Illumina OncoArray[1], comprising 533,631 SNPs. The remaining participants were genotyped with the Illumina iCOGS array, containing 211,155 SNPs[2]. Details about the quality control procedures and correlation between the arrays have been described previously[3-8]. European ancestry was determined using genetic data and multidimensional scaling. As previously published: "We excluded individuals of non-European ancestry using multi-dimensional scaling. For this purpose we selected 30,733 uncorrelated autosomal SNPs (pair-wise r2< 0.10) to compute the genomic kinship between all pairs of *BRCA1* and *BRCA2* carriers, along with 267 HapMap samples (CHB, JPT, YRI and CEU). These were converted to distances and subjected to multidimensional scaling. Using the first two components, we calculated the proportion of European ancestry for each individual and excluded samples with >27% non-European ancestry to ensure that samples of Ashkenazi Jewish ancestry were included in the final sample"[6]. Imputation of variants not on genotyping arrays was performed with IMPUTE2[9], after prephasing with SHAPEIT[10], using 1000 Genomes phase 3 as a reference panel. Imputation quality scores for the variants used in this study are shown in Table S2.

We used the 313-variant-based PRS for breast cancer developed in an independent study using data from the general population as described previously[11]; correlation between PRS based on the two genotyping arrays was high[8]. The PRS for overall breast cancer ($PRS_{313}$) and two ER-specific PRS, the ER-positive $PRS_{313}$ and ER-negative $PRS_{313}$ were calculated. For all three PRS, the same 313 variants were used for calculation with the following formula:

$$PRS_j = \sum_{i=1}^{313} n_{ij}\, w_i$$

In which $n_{ij}$ is the number of risk alleles (0, 1 or 2) for variant $i$ carried by individual $j$ and $w_i$ is the weight associated with variant $i$. All weights were derived from the analysis of data from the Breast Cancer Association Consortium (BCAC)[11]; for the ER-positive and ER-negative $PRS_{313}$, ER-specific weights were used for the subset of 116 variants with a significant difference in the effect size by subtype. The variants and their corresponding weights used in the PRS are listed in Table S2 as published previously[11]. The three PRS were standardized to the mean from all CIMBA participants,

including both unaffected and affected women, and to the SD in BCAC population controls which were included in the validation dataset[11]. The SDs used were 0.61, 0.65 and 0.59 for the $PRS_{313}$, ER-positive $PRS_{313}$ and ER-negative $PRS_{313}$ respectively. Using these SDs, the HR estimates for the associations of the standardized $PRS_{313}$ in our study are directly comparable with the OR estimates reported in the BCAC population-based study[11] and the HR estimates reported for primary breast cancer in *BRCA1* and *BRCA2* heterozygotes[7].

## Supplementary Figures

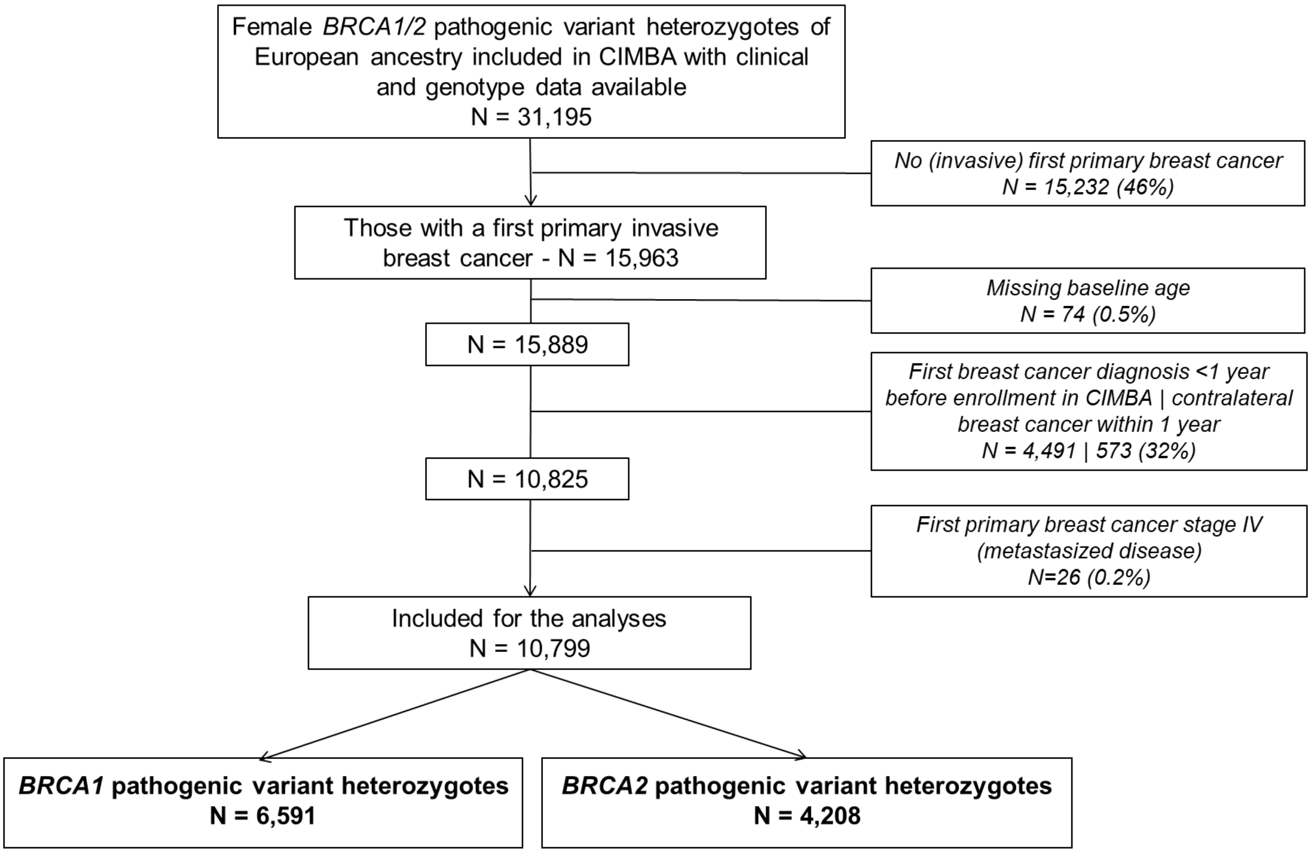


**Figure S1: Flow chart of the inclusion of CIMBA participants**

Flow chart of the inclusion and exclusion of CIMBA participants for this study.

Abbreviation: N, Number

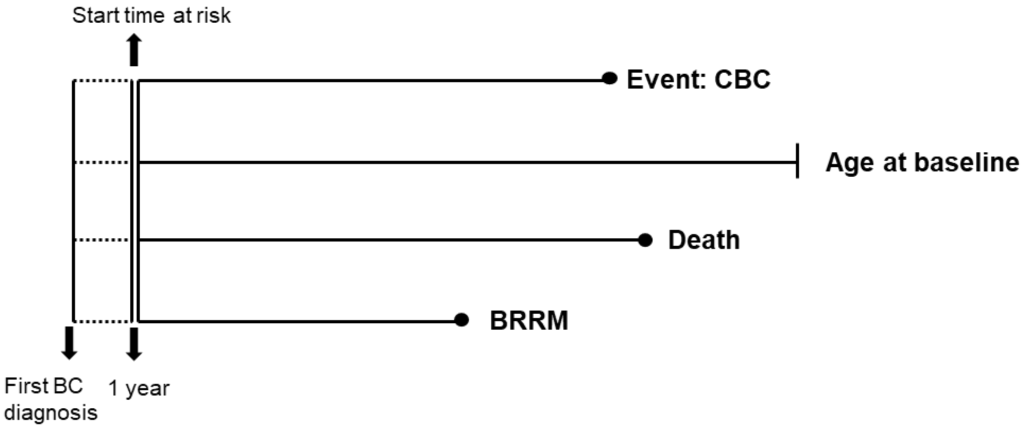


**Figure S2: Time at risk in the association analyses**

The time at risk was assumed to start one year after the first breast cancer. Participants were censored at (i) age at baseline, (ii) bilateral risk reducing mastectomy or (iii) death, whichever was earlier. Baseline age was defined as the age at local ascertainment (97%), or when this was not known, age at genetic testing (2%) or age at last follow-up (1%). Incidence of a metachronous contralateral breast cancer, invasive or *in situ*, before baseline was considered as an event in the main analyses.

Abbreviations: BC, Breast Cancer; BRRM, Bilateral Risk Reducing Mastectomy; CBC, Contralateral Breast Cancer.

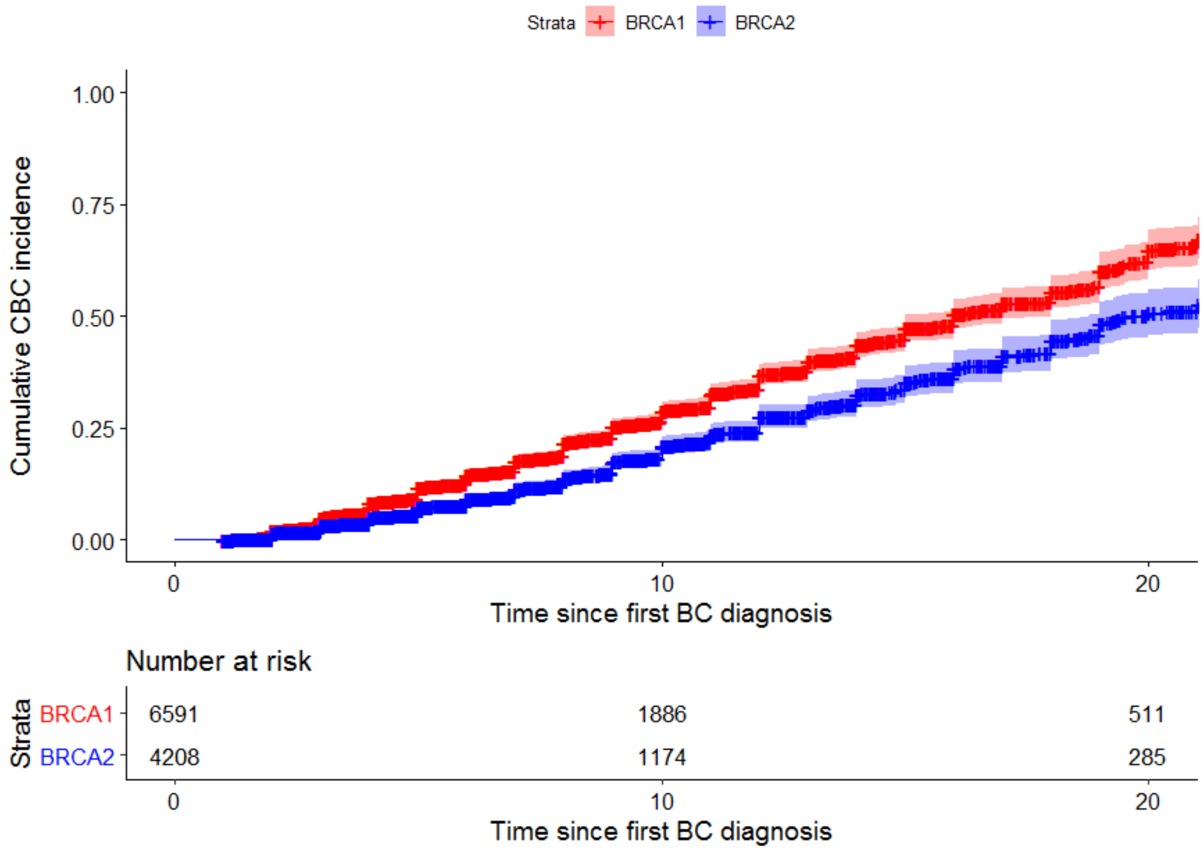


**Figure S3: Cumulative contralateral breast cancer incidence for *BRCA1* and *BRCA2* heterozygotes since the first breast cancer diagnosis**

Plot of the cumulative contralateral breast cancer incidence for *BRCA1* (red) and *BRCA2* (blue) pathogenic variant heterozygotes. Confidence intervals are shown with the transparent red and blue color. The time of follow-up started at the age of first primary invasive breast cancer diagnosis. Abbreviations: BC, Breast Cancer; CBC, Contralateral Breast Cancer.

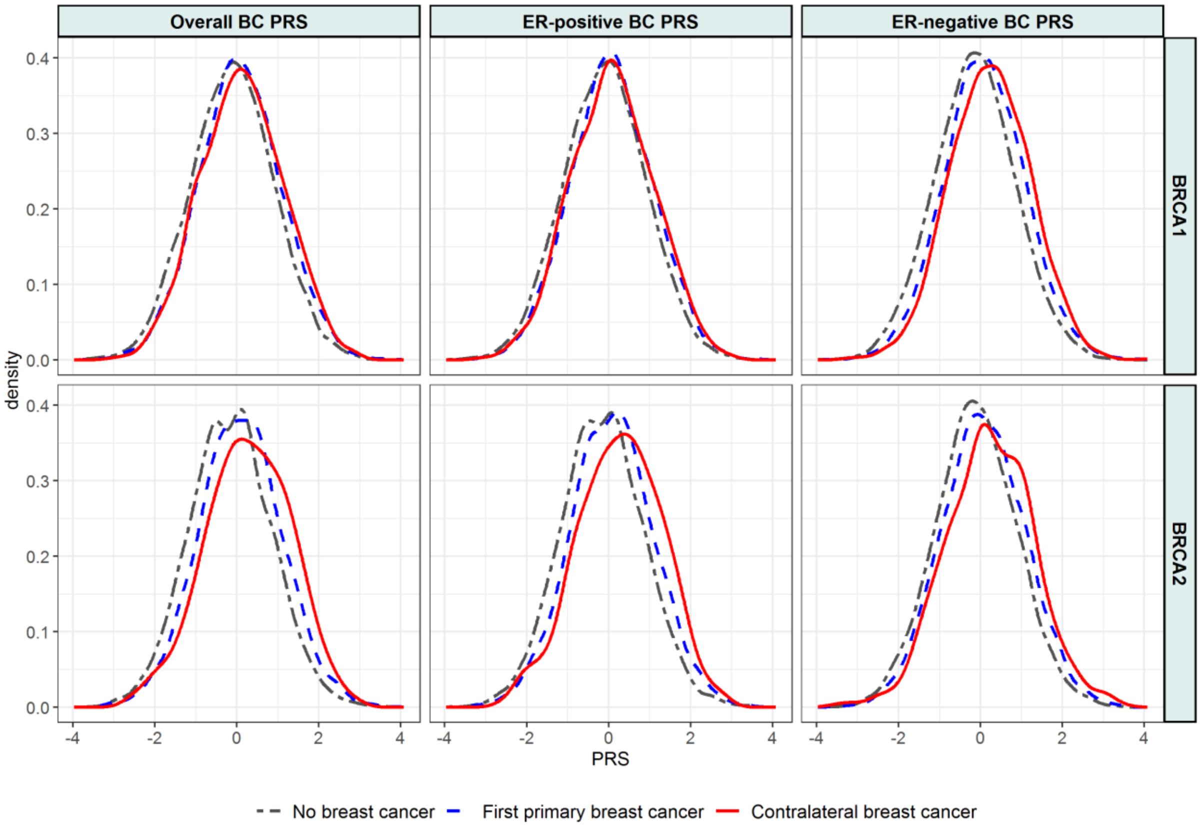


**Figure S4: Distribution of the overall breast cancer, ER-positive and ER-negative $PRS_{313}$ for *BRCA1* and *BRCA2* heterozygotes without breast cancer, with a first primary breast cancer and with contralateral breast cancer**

Density plots of the standardized PRS distributions for *BRCA1* and *BRCA2* heterozygotes. The distributions are shown for CIMBA participants who did not develop breast cancer (grey two-dashed line), who developed an invasive first primary breast cancer only (blue dashed line, selection shown in Figure S1) and who developed a metachronous contralateral breast cancer (red solid line). The number of included women for these groups were 8,837, 5,189, and 1,402 for *BRCA1* heterozygotes and 5,665, 3,561, and 647 for *BRCA2* heterozygotes.

Abbreviations: BC, Breast Cancer; ER, Estrogen Receptor; PRS, Polygenic Risk Score.

## Supplementary Tables

**Table S1: Estrogen receptor status of the first primary breast tumor and the contralateral breast tumor**

| | ER-status BC1 | ER-status CBC | | |
|---|---|---|---|---|
| | | ER-positive | ER-negative | Unknown |
| *BRCA1* heterozygotes | ER-positive | 25 | 42 | 25 |
| | ER-negative | 29 | 256 | 117 |
| | Unknown | 47 | 148 | 713 |
| *BRCA2* heterozygotes | ER-positive | 100 | 19 | 63 |
| | ER-negative | 16 | 18 | 27 |
| | Unknown | 81 | 13 | 310 |

Abbreviations: BC1, first primary Breast Cancer; CBC, Contralateral Breast Cancer; ER, Estrogen Receptor.

**Table S2: 313 variants included in the polygenic risk score**

First nine columns of the table were published by Mavaddat et al.[11]

**Table S3: Country of origin of included CIMBA participants**

| Country of origin | | *BRCA1* heterozygotes | *BRCA2* heterozygotes |
|---|---|---|---|
| Group[a] | Country | | |
| Africa | South Africa | 29 | 70 |
| America | Brazil | 0 | 1 |
| | Canada | 209 | 103 |
| | United States of America | 1266 | 735 |
| Asia | Israel | 60 | 52 |
| | Qatar | 0 | 1 |
| Australia | Australia | 355 | 269 |
| Eastern Europe | Albania | 1 | 0 |
| | Czech Republic | 41 | 0 |
| | Hungary | 120 | 36 |
| | Latvia | 9 | 0 |
| | Lithuania | 62 | 6 |
| | Poland | 217 | 0 |
| | Russia | 12 | 0 |
| Northwestern Europe | Austria | 179 | 77 |
| | Belgium | 128 | 43 |
| | Denmark | 224 | 171 |
| | Ireland | 1 | 1 |
| | Finland | 46 | 44 |
| | France | 677 | 565 |
| | Germany | 762 | 394 |
| | Iceland | 0 | 102 |
| | Netherlands | 440 | 196 |
| | Sweden | 177 | 24 |
| | United Kingdom | 702 | 614 |
| Southern Europe | Greece | 99 | 13 |
| | Italy | 472 | 285 |
| | Portugal | 23 | 58 |
| | Spain | 280 | 348 |

[a] Groups for country used in the cox-regression analyses

**Table S4: Results of the association analyses between the PRS and contralateral breast cancer risk**

| | | *BRCA1* heterozygotes | | | | | *BRCA2* heterozygotes | | | | |
|---|---|---|---|---|---|---|---|---|---|---|---|
| Outcome | $PRS_{313}$ | UBC cases, n | CBC cases, n | HR | 95% CI | P | UBC cases, n | CBC cases, n | HR | 95% CI | P |
| All CBC | Overall BC | 5,189 | 1,402 | 1.05 | 1.00 -1.11 | 0.059 | 3,561 | 647 | 1.15 | 1.07-1.24 | $2.33x10^{-4}$ |
| | ER-positive | | | 1.03 | 0.98-1.09 | 0.208 | | | 1.15 | 1.07-1.25 | $1.94x10^{-4}$ |
| | ER-negative | | | 1.12 | 1.06-1.18 | $5.98x10^{-5}$ | | | 1.11 | 1.03-1.20 | 0.005 |
| ER-positive CBC | Overall BC | 6,312[a] | 279[a] | 1.32 | 1.12-1.56 | 0.002 | 3,701[a] | 507[a] | 1.21 | 1.10-1.32 | $4.19x10^{-5}$ |
| | ER-positive | | | 1.30 | 1.11-1.52 | 0.002 | | | 1.22 | 1.11-1.33 | $2.15x10^{-5}$ |
| | ER-negative | | | 1.31 | 1.11-1.55 | 0.003 | | | 1.12 | 1.02-1.22 | 0.014 |
| ER-negative CBC | Overall BC | 5,468[a] | 1123[a] | 0.99 | 0.93-1.06 | 0.859 | 4,068[a] | 140[a] | 0.98 | 0.81-1.18 | 0.809 |
| | ER-positive | | | 0.98 | 0.92-1.04 | 0.491 | | | 0.95 | 0.79-1.15 | 0.628 |
| | ER-negative | | | 1.07 | 1.01-1.15 | 0.036 | | | 1.10 | 0.91-1.32 | 0.346 |

[a] Average number over 10 imputed datasets

Abbreviations: BC, Breast Cancer; CBC, Contralateral Breast Cancer; CI, Confidence Interval; ER, Estrogen Receptor; HR, Hazard Ratio; PRS, Polygenic Risk Score; UBC, Unilateral Breast Cancer.

**Table S5: Results of the change in effect size of the association between the PRS and contralateral breast cancer risk, using multivariable Cox Regression models**

| | | *BRCA1* heterozygotes; ER-negative $PRS_{313}$ | | | | | *BRCA2* heterozygotes; ER-positive $PRS_{313}$ | | | | |
|---|---|---|---|---|---|---|---|---|---|---|---|
| | Added variable | $β^a$ | % change | $HR^a$ | 95% CI | p | $β^b$ | % change | $HR^b$ | 95% CI | P |
| Base model[c] | | 0.111 | ref | 1.12 | 1.06-1.18 | $5.98x10^{-5}$ | 0.143 | ref | 1.15 | 1.07-1.25 | $1.94x10^{-4}$ |
| | Family history | 0.112 | 1.10 | 1.12 | 1.06-1.18 | $4.43x10^{-5}$ | 0.143 | 0.26 | 1.15 | 1.07-1.25 | $2.53x10^{-4}$ |
| | Age of BC1 | 0.112 | 1.03 | 1.12 | 1.06-1.18 | $4.32x10^{-5}$ | 0.151 | 5.01 | 1.16 | 1.08-1.26 | $1.29x10^{-4}$ |
| Tumor characteristics BC1 | ER-status | 0.111 | 0.04 | 1.12 | 1.06-1.18 | $4.28x10^{-5}$ | 0.141 | 1.68 | 1.15 | 1.07-1.24 | $3.73x10^{-4}$ |
| | Node status | 0.112 | 0.69 | 1.12 | 1.06-1.18 | $4.65x10^{-5}$ | 0.145 | 1.27 | 1.16 | 1.07-1.25 | $2.21x10^{-4}$ |
| | Tumor size | 0.111 | 0.01 | 1.12 | 1.06-1.18 | $5.36x10^{-5}$ | 0.147 | 2.24 | 1.16 | 1.07-1.25 | $1.95x10^{-4}$ |
| Therapy BC1 | Chemotherapy | 0.110 | 0.70 | 1.12 | 1.06-1.18 | $5.97x10^{-5}$ | 0.143 | 0.04 | 1.15 | 1.07-1.25 | $2.53x10^{-4}$ |
| | Hormone | 0.111 | 0.10 | 1.12 | 1.06-1.18 | $5.15x10^{-5}$ | 0.144 | 0.14 | 1.15 | 1.07-1.25 | $2.48x10^{-4}$ |
| | Trastuzumab | 0.111 | 0.02 | 1.12 | 1.06-1.18 | $5.22x10^{-5}$ | 0.143 | 0.23 | 1.15 | 1.07-1.25 | $2.57x10^{-4}$ |
| | Radiotherapy | 0.111 | 0.09 | 1.12 | 1.06-1.18 | $5.29x10^{-5}$ | 0.143 | 0.18 | 1.15 | 1.07-1.25 | $2.56x10^{-4}$ |
| Full model | All above variables combined | 0.114 | 2.24 | 1.12 | 1.07-1.18 | $4.50x10^{-5}$ | 0.150 | 4.37 | 1.16 | 1.07-1.26 | $2.06x10^{-4}$ |

[a] Effect size of the ER-negative $PRS_{313}$

[b] Effect size of the ER-positive $PRS_{313}$

[c] Cox regression model for the association between the PRS and contralateral breast cancer, stratified by country, clustered on family membership, and adjusted for birth cohort (quartiles of the observed distribution).

Abbreviations: BC1, first primary Breast Cancer; CI, Confidence Interval; HR, Hazard Ratio; PRS, Polygenic Risk Score

## References


1. Amos CI, Dennis J, Wang Z, et al. The OncoArray Consortium: A Network for Understanding the Genetic Architecture of Common Cancers. *Cancer epidemiology, biomarkers & prevention : a publication of the American Association for Cancer Research, cosponsored by the American Society of Preventive Oncology.* 2017;26(1):126-135.
2. Michailidou K, Hall P, Gonzalez-Neira A, et al. Large-scale genotyping identifies 41 new loci associated with breast cancer risk. *NatGenet.* 2013;45(4):353-352.
3. Gaudet MM, Kuchenbaecker KB, Vijai J, et al. Identification of a BRCA2-specific modifier locus at 6p24 related to breast cancer risk. *PLoS genetics.* 2013;9(3):e1003173.
4. Couch FJ, Wang X, McGuffog L, et al. Genome-wide association study in BRCA1 mutation carriers identifies novel loci associated with breast and ovarian cancer risk. *PLoS genetics.* 2013;9(3):e1003212.
5. Kuchenbaecker KB, Neuhausen SL, Robson M, et al. Associations of common breast cancer susceptibility alleles with risk of breast cancer subtypes in BRCA1 and BRCA2 mutation carriers. *Breast cancer research : BCR.* 2014;16(6):3416.
6. Milne RL, Kuchenbaecker KB, Michailidou K, et al. Identification of ten variants associated with risk of estrogen-receptor-negative breast cancer. *Nature genetics.* 2017;49(12):1767-1778.
7. Barnes DR, Rookus MA, McGuffog L, et al. Polygenic risk scores and breast and epithelial ovarian cancer risks for carriers of BRCA1 and BRCA2 pathogenic variants. *Genetics in Medicine.* 2020;22(10):1653-1666.
8. Kramer I, Hooning MJ, Mavaddat N, et al. Breast Cancer Polygenic Risk Score and Contralateral Breast Cancer Risk. *American journal of human genetics.* 2020;107(5):837-848.
9. Howie BN, Donnelly P, Marchini J. A flexible and accurate genotype imputation method for the next generation of genome-wide association studies. *PLoS genetics.* 2009;5(6):e1000529.
10. Delaneau O, Marchini J, Zagury JF. A linear complexity phasing method for thousands of genomes. *Nature methods.* 2011;9(2):179-181.
11. Mavaddat N, Michailidou K, Dennis J, et al. Polygenic Risk Scores for Prediction of Breast Cancer and Breast Cancer Subtypes. *American journal of human genetics.* 2019;104(1):21-34.
